# Precision Targeting Strategies in Pancreatic Cancer: The Role of Tumor Microenvironment

**DOI:** 10.3390/cancers16162876

**Published:** 2024-08-19

**Authors:** Nikolaos Vitorakis, Antonios N. Gargalionis, Kostas A. Papavassiliou, Christos Adamopoulos, Athanasios G. Papavassiliou

**Affiliations:** 1 Department of Biological Chemistry, Medical School, National and Kapodistrian University of Athens, 11527 Athens, Greece; nikolas.vitorakis@gmail.com; 2 Department of Clinical Biochemistry, ‘Attikon’ University General Hospital, Medical School, National and Kapodistrian University of Athens, 12462 Athens, Greece; agargal@med.uoa.gr; 3 First University Department of Respiratory Medicine, ‘Sotiria’ Hospital, Medical School, National and Kapodistrian University of Athens, 11527 Athens, Greece; konpapav@med.uoa.gr; 4 Department of Oncological Sciences, Icahn School of Medicine at Mount Sinai, New York, NY 10029, USA

**Keywords:** pancreatic ductal adenocarcinoma (PDAC), pancreatic cancer, tumor microenvironment (TME), cancer-associated fibroblasts (CAFs), myeloid-derived suppressor cells (MDSCs), tumor-associated macrophages (TAMs), immunotherapy

## Abstract

**Simple Summary:**

Pancreatic cancer is one of the most lethal forms of malignancies; therefore, new treatment strategies are required to increase the patients’ survival. It has been established that different cell types that surround pancreatic cancer cells, thus forming the tumor microenvironment, are responsible for tumorigenicity and inefficacy of treatments, including immunotherapy. In the present review, we aim to summarize current knowledge regarding the molecular mechanisms underpinning the interaction of cancer cells with cells of their microenvironment and discuss associated strategies to improve treatment results.

**Abstract:**

Pancreatic cancer demonstrates an ever-increasing incidence over the last years and represents one of the top causes of cancer-associated mortality. Cells of the tumor microenvironment (TME) interact with cancer cells in pancreatic ductal adenocarcinoma (PDAC) tumors to preserve cancer cells’ metabolism, inhibit drug delivery, enhance immune suppression mechanisms and finally develop resistance to chemotherapy and immunotherapy. New strategies target TME genetic alterations and specific pathways in cell populations of the TME. Complex molecular interactions develop between PDAC cells and TME cell populations including cancer-associated fibroblasts, myeloid-derived suppressor cells, pancreatic stellate cells, tumor-associated macrophages, tumor-associated neutrophils, and regulatory T cells. In the present review, we aim to fully explore the molecular landscape of the pancreatic cancer TME cell populations and discuss current TME targeting strategies to provide thoughts for further research and preclinical testing.

## 1. Introduction

Pancreatic cancer ranks among the top causes of cancer-related deaths globally, with its incidence having more than doubled in the past quarter century, from 196,000 cases reported in 1990 to 441,000 cases in 2017 worldwide [1]. Nearly all pancreatic tumors originate from ductal epithelial cells, developing as pancreatic ductal adenocarcinoma (PDAC), and are frequently associated with the expression of mutant *Kirsten rat sarcoma virus* (*KRAS*) gene, particularly the *KRAS*, *G12D*, *G12V*, and *G12R* variants [2]. Currently, PDAC ranks as the third most common reason for cancer-related deaths in the United States, with the potential to rise to the second position below lung cancer, thus surpassing colorectal cancer in the next decade [3,4]. Although this type of cancer represents approximately 2% of all cancer cases and is linked to 5% of deaths caused by cancer, our knowledge regarding its underlying molecular mechanisms is limited [5]. The likelihood of mortality due to pancreatic cancer increases significantly with age, rising from less than 2 deaths per 100,000 persons in a year among individuals aged 35–39 to over 90 among those aged over 80 years [1]. Several established risk factors include smoking, family history, alcohol consumption, chronic pancreatitis, diabetes, or the presence of a germline mutation [5]. It develops from noninvasive precancerous lesions, known as neoplasms, which are highly prevalent, and their occurrence increases with age [6]. While these lesions can progress to PDAC, the majority will not advance to cancer, and will thus not metastasize on their own [7]. Moreover, they can be cured if identified and treated promptly [8].

PDAC poses considerable clinical hurdles, mainly due to its late stage diagnosis and the development of therapeutic resistance. Delayed diagnosis often stems from the absence of symptoms in the early stages of the disease [5]. Even in advanced stages, non-specific symptoms such as fatigue, weight loss, anorexia, and abdominal or back pain, further complicate accurate diagnosis [9]. Less common symptoms may include steatorrhea, gastric outlet obstruction, acute pancreatitis, and venous thromboembolism [9]. Depending on the tumor’s location, symptoms differ, with tumors in the pancreatic body and tail causing pain, while those in the pancreatic head lead to jaundice and pruritus [6]. More than half of individuals with pancreatic cancer develop metastases, one-third have regional disease, and only one-tenth have localized disease [4]. Location of metastasis affects significantly PDAC prognosis, with liver metastasis being the most predominant and lung metastasis presenting the most favorable prognosis [10,11]. The median survival of a patient with advanced metastatic pancreatic cancer under treatment is typically one year, while it reduces to half without treatment. This is largely attributed to the advanced stage of the disease at the time of diagnosis, the restricted number of individuals deemed suitable for surgical resection, and the absence of chemotherapy regimens or other medications that can completely eradicate pancreatic cancer or provide a permanent cure [2,6]. Despite its ineffectiveness, cytotoxic chemotherapy represents the prevailing standard treatment for advanced or metastatic PDAC, offering a poor survival benefit [12].

Considering the limited efficacy of therapies in pancreatic cancer, a promising avenue for future progress involves focusing on the tumor microenvironment (TME) alongside immunotherapy as a combined approach [13]. Pancreatic cancer tumors exhibit a substantial desmoplastic stroma, comprised of diverse cellular components including pancreatic stellate cells, endothelial cells, and fibroblasts [13]. This stroma also encompasses a dense extracellular matrix (ECM), a poorly developed vascular system, and a heterogeneous population of immune cells, predominantly exhibiting suppressive phenotypes [14]. The TME is intricate and undergoes continuous evolution [15]. The cellular composition of PDAC tumors is predominantly non-cancerous, with neoplastic cells comprising a minority. Consequently, the disease’s aggressive characteristics, therapy resistance, and diversity are influenced to a considerable extent by the non-cancerous elements present within the TME, in addition to the PDAC cells themselves [14]. The diverse roles of these distinct cell populations result in varying nutrient needs and can lead to metabolic interactions that support tumor growth. These symbiotic relationships sustain PDAC metabolism, bolster resistance to chemotherapy, and contribute to immune suppression mechanisms. This substantial desmoplastic stroma hinders drug delivery and fosters an immunosuppressive microenvironment which is thought to be partially responsible for the ineffectiveness of chemotherapy and immunotherapy in PDAC [16,17]. In an aging society, the prevalence of individuals affected by pancreatic cancer will increase, highlighting the need for new and effective therapeutic targets such as the tumor microenvironment.

In this review, we thoroughly examined peer-reviewed articles available in English, found on the PubMed database, to explore how the tumor microenvironment impacts precision targeting methods in pancreatic cancer. In our search, we employed Boolean operators to merge essential terms like “pancreatic cancer”, “tumor microenvironment (TME)”, “cancer associated fibroblasts”, “precision targeting” and “therapeutic resistance”. We specifically focused on the molecular landscape of pancreatic cancer, the current standards of care and the potential of the TME as a target for personalized therapy.

## 2. Molecular Landscape of Pancreatic Cancer

Grutzmann et al. observed that alterations in gene expression in pancreatic cancer affected 568 genes, with 364 showing increased and 204 decreased expression [18]. Some of these genes are classified as driver genes that play essential roles in cancer development and progression, whereas others are passenger genes, which are not crucial for these processes [19]. The mutations detected in pancreatic cancer are mainly associated with oncogenes and tumor suppressor genes. The driver genes in PDAC are four: *KRAS*, *Small Mothers Against Decapentaplegic homolog 4* (*SMAD4*), *Cyclin-dependent kinase inhibitor 2* (*CDKN2A/p16*) and *Tumor suppressor protein 53* (*TP53*) [20]. In the oncogene category, *KRAS* mutations are prevalent in 80–95% of PDACs, particularly in Western individuals [21]. In cases where wild-type *KRAS* is detected, other genes, such as members of the RAS family or growth factor receptors, frequently assume the principal role in driving cancer. Examples of these genes are *BRAF*, *GNAS*, *FGFR2*, and *CTNNB1*, which have been recognized as alternative drivers of the disease [22,23,24,25]. When it comes to tumor suppressor genes, more than 90% of PDAC cases are characterized by *CDKN2A/p16* deactivation, followed by *TP53* downregulation found in six out of ten tumors, and *SMAD4* which is prevalent in half of the cases [26,27,28]. At the same time, roughly one in ten people (7%) with PDAC have germline mutations in the DNA repair genes, *BRCA2* and *MLH1*, whereas nearly 40% exhibit an overexpression of *EGFR* [29,30]. Researchers have also unearthed a collection of mutated genes, prevalent in less than one-fifth of PDAC patients. These genes, which are not considered traditionally primary drivers, include *RAC1*, *RNF43*, *ARID1A*, *ARID1B*, *TGFBR2*, *ROBO2*, *GATA6*, *ATM*, *SLIT2*, *MAP2K4*, *MAP3K21*, *SMARCA1*, *SMARCA4*, *MLL3*, *ACVR2A*, *ACVR1B*, *NRAS*, *PIK3CA*, *FAM133A*, *PBRM1*, *RBM10*, *ZMAT2*, and *KDM6A* [31,32,33,34,35]. In the initial phases, small tumors and preinvasive abnormalities show mutations in *KRAS*, *Her2*, and *Muc5* genes. Progressively, additional mutations appear in genes like *cyclin D1*, *CDKN2A*, *Muc5*, and *p16* during the intermediate stage, and in *BRCA2*, *DPC4/SMAD4*, and *TP53* genes in advanced stages [36,37]. In conjunction with minor genetic mutations in coding areas, research using innovative sequencing methods has been carried out to record substantial modifications in chromosomes. These encompass changes in the number of copies, rearrangements in chromosomes, and occurrences of chromothripsis [38]. While changes in DNA sequence are well-studied, different molecular alterations also play a role in PDAC development [39]. Epigenetic changes to the DNA and to histones can permanently alter the structure of chromatin and influence genetic activity [40]. Lastly, the excessive presence of microRNAs in pancreatic cancer is also believed to be involved in the development of pancreatic tumors [41].

Pancreatic cancer stands as one of the most formidable challenges in oncology, marked by its aggressive nature and limited treatment options. Within the intricate landscape of pancreatic cancer development and progression, a multitude of signaling pathways orchestrate the cellular mechanisms underlying tumorigenesis. Among these, the Nrf2, AMPK, MAPK, PI3K/Akt, STAT3, Wnt/β-catenin, non-coding RNAs, and NF-κB pathways emerge as pivotal players, each offering unique insights into the disease’s pathophysiology and therapeutic potential. Nrf2, renowned for its role in cellular defense against oxidative stress, assumes an oncogenic function in the context of pancreatic cancer, intricately linked to processes like apoptosis, mRNA translation, and metabolism modulation [42,43]. Elevated Nrf2 expression fosters a pro-tumorigenic microenvironment through enhanced antioxidant capacity and metabolic reprogramming, while also promoting tumor cell survival and metastasis [44]. Notably, targeting Nrf2 holds promise in overcoming chemotherapy resistance and sensitizing PC cells to conventional therapies [45,46]. Conversely, AMPK emerges as a tumor suppressor, exerting its influence through autophagy induction, metabolic regulation, and inhibition of proliferation [47,48]. MAPK pathways, on the other hand, contribute to both carcinogenesis and metastasis in pancreatic cancer [49,50]. Dysregulation of MAPK signaling pathways fuels tumor growth and dissemination, underscoring the need for targeted interventions to eliminate its oncogenic effects. The PTEN/PI3K/Akt axis introduces a complex interplay between tumor-suppressive and tumor-promoting elements, wherein PTEN exerts inhibitory effects counteracted by the pro-tumorigenic actions of PI3K/Akt [51]. Dissecting this intricate balance unveils potential therapeutic avenues, particularly in circumventing chemoresistance and modulating the TME to enhance treatment outcomes [52,53]. STAT3 has emerged as a crucial regulator in the pathogenesis of pancreatic cancer, driving key cellular processes that facilitate tumor progression [54]. Targeting STAT3 with inhibitors offers a promising strategy to overcome therapeutic resistance and curb metastatic spread, presenting an exciting avenue for precision therapy in pancreatic cancer. Although preliminary clinical trials had shown promising results in the case of JAK/STAT pathway inhibitor napabucasin, it later failed to increase overall patient survival in two phase III clinical trials [55]. Additionally, a combinatorial treatment with an antisense STAT3 inhibitor, AZD-1950, and an anti-PD-L1 monoclonal antibody, durvalumab, has advanced to a phase II clinical trial which includes advanced refractory pancreatic cancer patients (NCT02983578) (Table 1), with minimal toxicity being reported [2]. The Wnt/β-catenin pathway emerges as a key driver of pancreatic cancer metastasis and stemness, exerting its oncogenic effects through epithelial-to-mesenchymal transition (EMT) induction and metabolic reprogramming [56]. Non-coding RNAs (ncRNAs) unveil a vast regulatory landscape, encompassing microRNAs (miRNAs), long non-coding RNAs (lncRNAs), and circular RNAs (circRNAs) [57]. These elusive molecules orchestrate a myriad of cellular processes, from apoptosis and proliferation to immune modulation and chemoresistance [58]. Harnessing the therapeutic potential of ncRNAs holds promise in reshaping the treatment paradigm for pancreatic cancer, offering novel avenues for precision medicine [59]. Lastly, NF-κB emerges as a central player in orchestrating the inflammatory milieu within the pancreatic cancer microenvironment, mediating immune evasion, and chemoresistance [60].

## 3. Current Standards of Care

As mentioned before, pancreatic cancer is still one of the most complicated malignancies to treat, often with a late-stage diagnosis which limits the available options. Current treatment strategies mainly involve surgery in combination with chemotherapy and radiation, according to every patient’s specific condition. Targeted therapies and immunotherapy seem promising, but further research is essential to improve survival rates.

The treatment and outlook for patients with pancreatic cancer largely depend on whether the tumor can be surgically removed. Patients with partial tumor removal have survival rates similar to those of patients with metastases [4]. It is therefore evident that although there is still confusion regarding the classification of tumors as resectable or borderline resectable, health professionals should be meticulous in identifying patients who can undergo complete, curative surgery [61]. The preferred treatment for resectable pancreatic tumors, without metastases or invasion of blood vessels, typically involves surgically removing the site of the tumor in combination with adjuvant chemotherapy. Surgical options are pancreaticoduodenectomy (the Whipple procedure), where the pancreatic head is surgically extracted, distal pancreatectomy for the tail, or total pancreatectomy for extensive involvement [6]. The 5-year survival rate after surgical intervention remains notably low, standing lower than one in five patients [2]. Nonetheless, the implementation of specific adjuvant chemotherapy protocols has demonstrated a potential for modestly augmenting survival outcomes [17]. Recent development in this field is the use of laparoscopic surgery in almost 1 in 7 patients [4]. The choice of subsequent treatment options for patients experiencing recurrence after resection depends on where the recurrent disease is located. Most patients with recurrent disease progress to metastasis, warranting consideration of systemic chemotherapy. If the site of the metastasis is the liver, surgical removal can enhance survival outcomes. Particularly, in the case of patients with compromised performance status, palliative care and comprehensive supportive interventions stand as feasible approaches [16].

Despite surgical resection, 75% of patients recur within two years, suggesting micro-metastatic disease, thus necessitating post-operative adjuvant chemotherapy. The adjuvant chemotherapy is usually administered one to two months post-surgery for six months according to NCCN and ASCO [62]. The choice of treatment regimen for each patient may differ depending on their health condition and the stage of cancer [6]. Currently, some of the adjuvant treatment modalities include revised FOLFIRINOX, gemcitabine combined with cisplatin, gemcitabine with nab-paclitaxel, and single-agent gemcitabine or fluorouracil [63]. Additionally, S-1, an oral prodrug of 5-fluorouracil, is used [13]. Gemcitabine works by producing dFdCDP, inhibiting DNA synthesis, but its modest survival benefit is hindered by the TME [64]. On the other hand, 5-Fluorouracil (5-FU) disrupts RNA and DNA by incorporating nucleotides and inhibiting thymidylate synthase, inducing severe genomic damage in cancer cells [2]. A Cochrane database analysis suggests that combining multiple chemotherapy agents in advanced pancreatic carcinoma is more effective than a single treatment, despite increased adverse reactions such as sensory neuropathy, fatigue, gastrointestinal problems, and neutropenia [65]. Modified FOLFIRINOX, which is primarily offered post-surgery to suitable patients, combines 5-FU, irinotecan, oxaliplatin, and leucovorin, demonstrating a six-month progression-free survival among patients with pancreatic cancer [66]. Combination therapies involving gemcitabine are effective for individuals unsuitable for FOLFIRINOX, while single-agent treatment is administered to those unable to tolerate any combination therapies [16]. Individuals eligible for additional combination therapy would receive an alternative second-line treatment regimen. For example, those previously treated with fluoropyrimidine-based therapy might be prescribed gemcitabine combined with albumin-bound (nab) paclitaxel. Conversely, patients previously treated with gemcitabine may undergo FOLFIRINOX or a modified 5-FU-based regimen such as FOLFIRI or FOLFOX.

According to Gugenheim et al. there are two types of preoperative therapies [67]. In the case of patients with resectable pancreatic cancer, neoadjuvant therapy is used to optimize surgical outcomes through increasing the likelihood of margin-negative resections (R0), whereas in the case of patients with initially unresectable pancreatic cancer conversion, or in other words downsizing therapy, is implemented to make inoperable tumors resectable [67]. In both cases, the patient’s overall survival is improved. In resectable pancreatic cancer (RPC), in borderline resectable pancreatic cancer (BRPC), and in locally advanced pancreatic cancer (LAPC), therapy primarily includes multi-drug chemotherapy regimens like FOLFIRINOX and gemcitabine-based drugs often combined with radiotherapy [68]. While upfront surgery is still the standard, neoadjuvant therapy is being explored for high-risk patients. For borderline resectable pancreatic cancer (BRPC), FOLFIRINOX and gemcitabine with nab-paclitaxel are commonly used, often combined with radiotherapy. If patients respond well, surgery is possible, though often requiring vascular resection, to achieve complete removal [4,6,69]. Unfortunately, these treatments have limited efficacy since typically less than 40% become eligible for surgery after such treatment [70].

The management of non-resectable and metastatic disease differs significantly between patients. Intra-abdominal metastases are generally not recommended for resection. In contrast, if there are secondary tumors that develop in the lungs following the treatment of the primary cancer, they may be considered for resection, leading to increased survival [4]. Typically, the initial treatment for most cases involves FOLFIRINOX or gemcitabine combined with albumin-bound (nab) paclitaxel. Apart from nab-paclitaxel, nano liposomal irinotecan (NAL-IRI) is also utilized in MM-398, an innovative nanoliposomal version of irinotecan, and in NALIRIFOX, which combines NAL-IRI with 5-fluorouracil (5-FU) and leucovorin (LV). Its advantageous pharmacokinetic properties allow it to enhance tumor targeting while reducing overall toxicity [69]. According to the findings of the NAPOLI-1 research in 2015, the combination of 5-fluorouracil and leucovorin (5-FU/LV) with nano liposomal irinotecan (nal-IRI) received FDA approval as a second-line treatment for individuals with metastatic PDAC following gemcitabine-based treatment, whereas after the NAPOLI 3 was completed NALIRIFOX was approved as a first-line treatment for metastatic PDAC as well [71]. In general, patients treated with nano liposomal irinotecan (NAL-IRI) experienced extended survival and delayed cancer progression in comparison to those who received gemcitabine plus (nab) paclitaxel [72]. Patients with diminished performance status may receive solitary treatment regimens. As mentioned above, fluoropyrimidine-based regimens are second line after gemcitabine and vice versa. Emerging studies highlight the benefits of maintenance therapy to certain patients. In 2019, Olaparib garnered approval as a maintenance regimen for individuals harboring *BRCA1* or *BRCA2* mutations and metastatic disease [6].

Lastly, in pancreatic cancer cases, palliative care is always an available option. Whether as a primary treatment approach or alongside curative treatments, palliative care focuses on symptom management, pain relief, and improving overall quality of life for patients facing this challenging diagnosis [16].

## 4. Targeting Pancreatic Tumor Microenvironment (TME)

Despite the application of combinational chemotherapies, metastatic PDAC remains among the deadliest solid tumors. These dismal survival rates have driven continuous efforts to target the tumor microenvironment (TME) for therapeutic purposes. Ongoing studies focusing on genetic alterations in the TME propelling advancement and spread of pancreatic cancer are revealing specific pathways that can be targeted in certain groups of patients. Early identification of these groups during diagnosis can provide the potential for tailored therapy and improve the treatment prognosis. Advancements in our understanding of the pathways involved in PDAC progression offer the potential to devise novel pharmaceuticals, consequently enhancing treatment results for most patients. In this section, we will analyze the main components of the TME, their contribution to pancreatic carcinogenesis, and finally, their potential as therapeutic targets.

**Figure 1:**
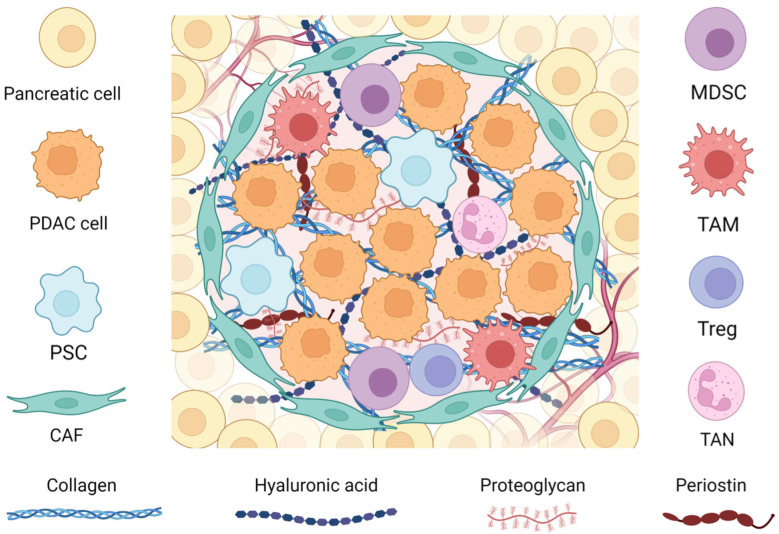
Pancreatic tumor microenvironment. CAFs, cancer-associated fibroblasts; MDSCs, myeloid-derived suppressor cells; PDAC, pancreatic ductal adenocarcinoma; PSCs, pancreatic stellate cells; TAMs, tumor-associated macrophages; TANs, tumor-associated neutrophils; Tregs, regulatory T cells.

The TME represents a complex and dynamic ecosystem of cells and extracellular components that co-develop with cancer cells, offering essential support [73]. The intricate pathways through which the cells of the TME interact are yet to be completely understood and deciphered. Major progress in this field was achieved with the use of Spatial Analysis Techniques CyTOF and scRNA-seq in PDAC cells, highlighting the diverse TME nature [14]. In physiological conditions, every tissue can achieve homeostasis in the case of an imbalance through the assistance of the connective, vascular, and immune constituents of its surrounding stroma [74]. With the development of cancer, these natural reactions are altered since cancer through the KRAS pathway co-opts them to establish a supportive TME conducive to its thriving expansion [75,76]. Pancreatic cancer, in other words, presents with a complex stroma consisting of a variety of cancerous and non-cancerous cells as well as their products. These include a wide variety of fibroblasts, a compact ECM, suppressive immune cells, an ill-defined vascular system, pancreatic myofibroblast-like cells (PSCs), signaling molecules, and proteins [77]. Out of these components, there is a clear association between cancer-associated fibroblasts (CAFs), regulatory T cells (Tregs), pancreatic stellate cells (PSCs), tumor-associated macrophages (TAMs), and myeloid-derived suppressor cells (MDSCs) with cancer advancement [78] (Figure 1). This stroma, which has an extensive fibrotic character due to PSCs, can make up a considerable proportion of the entire bulk of the tumor, accounting for as much as 90% of its volume [79]. Extensive fibrosis, desmoplasia, is observed in both primary and metastatic tumors, but their TME differs. This discrepancy is influenced by factors such as the tumor’s location and whether the patient has undergone therapy, which can alter tumor programming [80]. The understanding now is that the impact of the non-cancerous elements of the TME on aggressiveness, therapy resistance, and diversity of PDAC is as significant as that of the cancerous cells.

As mentioned above, the TME comprises a complex array of cell types pivotal to disease progression with key players including CAFs, PSCs, a diverse repertoire of immune cells as well as the ECM (Figure 1). In this section, we will provide a concise analysis of its principal cellular components.

The ECM in the TME plays a crucial role in the progression and behavior of PDAC. It constitutes an intricate network comprising structural elements, enzymes, scaffold proteins, and proteoglycans, all of which collectively uphold biochemical and structural equilibrium [81]. ECM’s production is enhanced in the case of pancreatic cancer both in primary and metastatic sites, with the predominant components being hyaluronic acid (HA), collagens type I, III, and IV [2,82,83]. Signaling between neighboring cells in the tumor stroma in the case of pancreatic cancer prompts a desmoplastic reaction by inducing stromal fibroblasts to increase their production of fibronectin and collagen [84]. Specific elements of the ECM have been observed to associate with median patient survival in PDAC. Studies have established a correlation indicating that lower levels of stromal HA and collagen are linked to improved survival rates, particularly when type I collagen is present. Conversely, higher levels of these components tend to be associated with less favorable outcomes. However, additional experiments are necessary to substantiate these findings [83,85]. This indicates that collagens play an active role in PDAC beyond structural support, influencing cancer cell proliferation, metastasis, and, in turn, survival.

### 4.1. Pancreatic Stellate Cells

**Figure 2:**
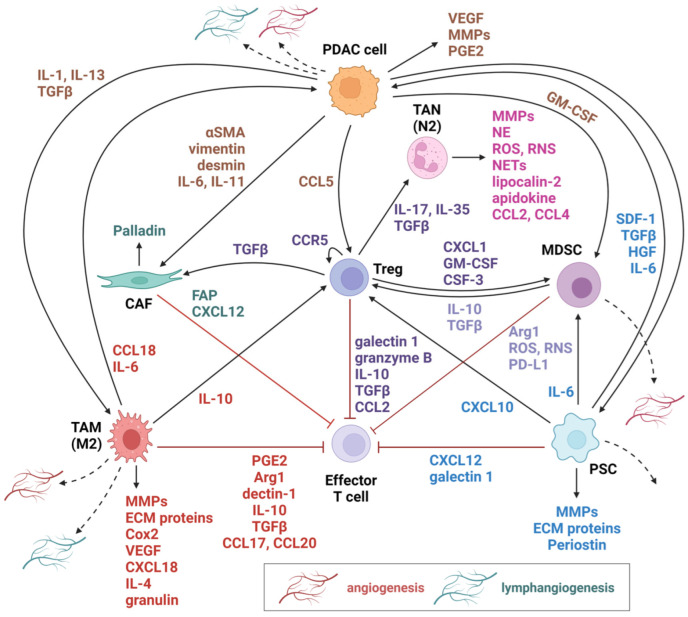
This figure illustrates the complexity of cellular interactions among TME cells in PDAC. These interactions form a network that helps the tumor grow, evade from the immune system, and spread somewhere else in the body. The main players of this landscape are represented by CAFs, TAMs (M2 type), TANs (N2 type), Tregs, effector T cells, MDSCs, and PSCs. All the abovementioned cells communicate with PDAC cells through the production of several chemokines and cytokines which regulate tumor growth, progression as well as metastasis through angiogenesis and lymphangiogenesis. This complex interconnected network determines the development and behavior of PDAC. TME, tumor microenvironment; PDAC, pancreatic ductal adenocarcinoma; CAFs, cancer-associated fibroblasts; TAMs, tumor-associated macrophages; TANs, tumor-associated neutrophils; Tregs, regulatory T cells; MDSCs, myeloid-derived suppressor cells; PSCs, pancreatic stellate cells; IL-1/4/6/10/11/13/17/35, interleukin-1/4/6/10/11/13/17/35; TGFβ, transforming growth factor β, VEGF, vascular endothelial growth factor; MMPs; matrix metalloproteinases; PGE2, prostaglandin E2; GM-CSF, granulocyte–macrophage colony-stimulating factor; αSMA, α smooth muscle actin; CCL-2/4/5/17/20, C-C motif chemokine ligand -2/4/5/17/20; NE, neutrophil elastase; ROS, reactive oxygen species; RNS, reactive nitrogen species; NETs, neutrophil extracellular traps; CCR5, C-C chemokine receptor type 5, CXCL-1/10/12/18; C-X-C motif chemokine ligand-1/10/12/18; ECM, extracellular matrix; Cox2, cyclooxygenase-2; CSF-3, colony-stimulating factor 3; FAP, fibroblast activation protein; SDF-1, stromal cell-derived factor 1; HGF, hepatocyte growth factor; Arg1, arginase 1; PD-L1, programmed death-ligand 1.

Pancreatic Stellate Cells (PSCs) are myofibroblast-like cells predominantly situated within the exocrine pancreatic areas [86]. Their principal function revolves around fostering the establishment of desmoplasia in pancreatic cancer. A variety of molecular pathways regulate PSCs’ ability to initiate desmoplasia such as the CXCL12/CXCR4, IL-6, the vitamin D receptor (VDR), and paracrine sonic hedgehog (SHH) [87]. One of the extensively studied pathways involves KRAS triggering SHH expression via nuclear factor-κB (NF-κB), subsequently activating GLI1 in the stroma to promote a microenvironment conducive to tumor growth [88,89]. In addition to promoting desmoplasia, PSCs exhibit immunosuppressive properties by producing molecules like CXCL12 and galectin-1 (Figure 2). These compounds induce the production of Th2 cytokines, impede the migration of CD8+ T cells into the tissue surrounding the tumor, and promote T cell apoptosis [90,91]. PSCs can also attract Tregs and stimulate the maturation of MDSCs in the TME to bolster their immunosuppressive role [92]. PSCs also advance cancer by synthesizing molecules like galectin-1, hepatocyte growth factor (HGF), stromal cell-derived factor-1 (SDF-1), IL-6, and TGFβ [93]. PSCs can be categorized as either quiescent (qPSCs) or activated (aPSCs) [78] (Figure 2). The first category synthesizes MMP-2, MMP-9, and MMP-13 along with their inhibitors, as well as stores vitamin A, collectively contributing to the maintenance of physiological tissue integrity [94]. Quiescent PSCs can undergo activation in response to environmental stresses like HIF1α, IL-1, TGFβ, and IL-6, transitioning into a myofibroblast-like phenotype. This transformation creates a conducive microenvironment for tumor growth within the tissue [78,87]. These PSCs exhibit reduced cytoplasmic lipid droplets, elevated MMPs, and ECM protein expression, and increased ability to proliferate [86]. Furthermore, they play a crucial role in the pancreatic cancer environment by releasing factors like SDF-1, HGF, galectin-1, TGFβ, and IL-6, promoting pancreatic cancer progression [93] (Figure 2). It is vital to note that activated pancreatic stellate cells (aPSCs) are not indefinitely maintained as they can return to their quiescent state due to cell death, aging, tissue decline or regeneration [95].

### 4.2. Cancer-Associated Fibroblasts

Cancer-Associated Fibroblasts (CAFs) are the predominant cell type in the tumor microenvironment, mainly tasked with arranging collagen fibrils to induce EMT and depositing the extracellular matrix, thus creating routes for cancer invasion [2,96]. Tumor necrosis factor (TNF-α), IL-1, IL-6, IL-10, TGF-β, and the sonic hedgehog (SHH) pathway are only a few of the variant cytokines and pathways involved in CAFs activation [97]. The complete functions of CAFs in pancreatic cancer progression remain incompletely understood. So far, they have been seen solely as promoters of tumor growth. By secreting a range of growth factors and other signaling compounds such as α-smooth muscle actin (αSMA), vimentin, and desmin, these CAFs actively promote cancer progression [98,99]. Research using mouse models has demonstrated that CAFs develop drug resistance by producing cytokines, altering the ECM structures, developing PDAC-promoting metabolic pathways, and triggering epigenetic and genetic changes [82,100]. Based on these findings, studies have concentrated on inhibiting signaling pathways that activate CAFs, particularly the SHH pathway. However, certain PDACs exhibited even further progression following this strategy [101,102]. Through similar experiments, alongside the identification of CD105-negative CAFs known to impede cancer progression, it has been evidenced that diverse CAF subtypes exist, each exerting specific influences on PDAC, either fostering or restraining its growth [103]. The distinct types of CAFs include the myofibroblast-like ones (myCAF), situated near the cancerous cells, primarily tasked with ECM formation, and the inflammatory fibroblast ones (iCAF), which reside among clusters of PDAC cells, secreting anti-inflammatory proteins like IL-6 [104]. In this way, myCAFs are driven by TGFβ to generate the surrounding stroma while iCAFs promote tumor proliferation [74]. Apart from myCAFs and iCAFs, antigen-presenting CAFs, a recently identified molecule, express MHCII and engage in antigen presentation to T cells. Furthermore, it has been postulated that CAFs may induce immunosuppression by promoting the exclusion of T cells. This speculation arises from the identification of CAFs producing fibroblast activation protein (FAP) and CXCL12, which inhibit the infiltration of T cells into the tumor microenvironment [105,106]. Besides the diversity among CAFs, their phenotype and contribution to cancer advancement can be shaped by gene mutations of genes and their spatial distribution. CAF types and their arrangement within the tumor differ across different areas, forming unique “sub-tumor microenvironments” among patients, indicating personalized features of the TME [107,108].

### 4.3. Tumor-Associated Macrophages

In the TME of PDAC, a diverse array of immune cells orchestrates a complex interplay that profoundly influences disease progression and treatment outcomes. Firstly, TAMs are classified into two distinct subtypes based on their functional characteristics. M1 macrophages predominantly secrete interleukin-12 (IL-12) and exhibit anti-tumor properties, while M2 macrophages primarily produce interleukin-10 (IL-10) and promote desmoplasia, immunosuppression and, in turn, cancer progression through the activation of the PI3K*γ* pathway [109]. Their capacity to induce desmoplasia arises from their interaction with PSCs, whereas their immunosuppressive properties stem from their production of compounds such as PGE2, dectin-1, Arg1, TGFβ, IL-10, CCL17, and CCL20, which enhance Tregs while concurrently inhibiting CD8+ T cells (Figure 2) [110,111,112]. Their immunosuppressive properties are enhanced by substances like PD-L1 which are expressed on TAMs’ surface and lead to apoptosis of T-cells [113]. TAMs also secrete proteases like matrix metalloproteinases (MMPs), ECM proteins, and angiogenic factors such as Cox2 and VEGF, all of which contribute to the enhancement of metastasis [114]. Finally, TAMs produce IL-6, which triggers activation of the JAK/STAT3 pathway, thereby worsening early lesions and fostering carcinogenesis [115].

### 4.4. Tumor-Asociated Neutrophils

In contrast to TAMs, the biological function of tumor-associated neutrophils (TANs) in PDAC remains less well-defined. Nevertheless, TANs’ presence in the TME is linked to unfavorable prognostic outcomes across various cancers (Figure 2) [116]. TANs are divided into two distinct subpopulations, the N1 and N2. This separation occurs by TGFβ and IFN*α*, respectively. N1 depict pro-inflammatory features which recruit and activate CD8+ cells. N2 favor tumor promotion and TME remodeling through the secretion of MMPs, CCL2/3/4/17, neutrophil elastase, and reactive oxygen species (ROS). Genes such as *VEGFA*, *PLAU*, *LGALS3*, *PDE4D*, and *LDHA* are upregulated. *PLAU*, specifically, plays a crucial role in encoding urokinase plasminogen activator, which acts as an upstream activator of the cMET pathway in cancer cells. The production of neutrophil extracellular traps (NETs) by IL-17 enhances the development of liver metastasis and resistance to immunotherapy by suppressing CD8+ cells [117,118].

### 4.5. Myeloid-Derived Suppressor Cells

The TME in PDAC is additionally marked by the presence of myeloid-derived suppressor cells (MDSCs), which migrate from the bone marrow under the influence of cytokines like GM-CSF [119]. Their main function in this context is immunosuppression, achieved through various pathways that inhibit the activities of effector T cells. Firstly, MDSCs generate ROS, minimizing antigen-dependent proliferation in T cells by preventing the translation of the CD3ζ chain (Figure 2). In MDSCs, activation of the STAT3 pathway induces the expression of both Arg1 and inducible nitric oxide synthase (iNOS), which results in upregulation of PD-L1. STAT3 also reduces L-arginine levels in the TME, thereby impairing T cell activation and proliferation [120]. A specific type of MDSCs, Polymorphonuclear MDSCs, have antigen-presenting capabilities enhancing immune tolerance and aiding immune escape [121]. Finally, experimental studies conducted in vitro have underscored the immunosuppressive role of MDSCs, revealing their capacity to foster regulatory T cell development.

### 4.6. Regulatory T Cells

Regulatory T cells (Tregs) play a crucial role in PDAC by inhibiting effector T cells and fostering an immunosuppressive environment around the tumor. They are drawn to the tumor site by compounds such as CCR5, which they produce, thereby creating a positive feedback loop that further enhances the tumor’s immunosuppressive milieu [122]. Tregs employ multiple pathways to inhibit the immune response against tumors. They release compounds, including galectin-1, granzyme B, CCL2, IL-10, and TGFβ, all of which contribute to the inhibition of effector T cells [77,123] (Figure 2). They engage the TRAIL pathway and competitively bind IL-2, depriving effector cells of this crucial growth factor [124]. Furthermore, Tregs emit CXCL1, GM-CSF, and CSF-3, facilitating the accumulation of MDSCs and further fortifying the tumor’s immune evasion mechanisms (Figure 2) [78]. FOXP3+ Tregs also activate the TRAIL pathway and produce many TGFβ ligands, which induce the differentiation of CAFs, thereby facilitating tumor progression [125,126].

## 5. Pancreatic Cancer Progression and Resistance: The Role of the Tumor Microenvironment

The development and persistence of cancer’s defining characteristics, including continuous cell growth, evasion of cell death, promotion of blood vessel formation, facilitation of invasion, and spread to distant sites, stimulation of inflammatory processes that support tumor growth, and evasion of immune system detection and destruction, are influenced to differing extents by factors within the TME. This expansive and diverse terrain presents numerous obstacles to the effectiveness of therapies. PDACs are often hypo-vascularized tumors since the dense desmoplasia surrounding them traps and deactivates molecules that promote the growth of new blood vessels [127]. Hypoxia exacerbated by the presence of antiangiogenic factors plays a significant role in the aggressiveness of pancreatic cancer, inducing metabolic changes, inhibition of cell death, continuous cell growth, resistance to treatment, invasion, and spread to distant sites [128]. In more detail, reduced vascularity inhibits the ability of drugs to permeate and spread within the tumor microenvironment, thereby restricting their effectiveness. Inadequate blood supply also creates a nutrient-poor environment that triggers cancer cell autophagy to sustain metabolic functions and tumor growth. This condition is also associated with heightened resistance to cytotoxic therapies, as well as immune evasion by breaking down the MHC-I in PDAC cells, which is essential for their recognition by cytotoxic T cells [129]. Cancer cells also trigger autophagy in stromal cells, which serve as a source of energy for them [130]. Fibrosis, frequently observed in PDAC, serves as a physical obstacle to the effectiveness of treatments. CAFs aid in fibrosis by generating elements of the ECM and altering the stromal environment by facilitating collagen cross-linking. This process adjusts tumor rigidity, thereby promoting tumor advancement and the migration of cancer cells [96]. Another notable feature of PDAC is its significant resistance to immunotherapy, primarily due to its immunosuppressive or “cold” microenvironment. Cancer cells might escape detection by the immune system due to their limited antigenicity and the absence of neoantigens, rendering them less immunogenic [131]. Moreover, they inhibit the infiltration of immune cells. Pancreatic cancer cells effectively hinder the activation of CD8+ T cells while increasing the number of regulatory immune cells. This involves reducing the expression of MHC I and lowering Fas expression, which facilitates the cells’ ability to resist apoptosis [77]. Additionally, they release immunosuppressive cytokines like TGFβ and IL10, contributing to immune suppression within the TME [132]. PDAC is known for drawing in myeloid and fibroblast populations with characteristics that suppress the immune system and promote tumor growth [133]. Pancreatic cancer also often coincides with systemic inflammation, as indicated by an increased ratio of neutrophils to lymphocytes, which correlates with decreased effectiveness of cytotoxic chemotherapy [134]. Finally, pancreatic cells produce the programmed cell death protein-1 ligand (PD-L1), cytotoxic-T-lymphocyte-associated protein 4 (CTLA-4), Forkheadbox protein 3 (Foxp3), and Indoleamine 2,3-dioxygenase (IDO) [77]. These compounds induce a state of reduced responsiveness in anti-tumor T cells, bolster the function of Treg cells via the generation of CCL5, and negatively impact the activity of natural killer (NK) cells [135,136].

The TME also significantly contributes to cancer metastasis and by default to a worse survival prognosis for the patient. The main processes driving metastasis encompass the formation of new blood and lymphatic vessels, the EMT transition, penetration into adjacent tissues, cellular migration, creation of a pre-metastatic niche, and eventual proliferation at the distant metastatic location. Pancreatic cancer cells, alongside cells like MDSCs and TAMs found in the TME, stimulate the development of new vascular networks to support tumor blood flow and promote metastasis. They accomplish this by secreting a variety of factors that promote angiogenesis, including pro-angiogenic proteins, growth factors and cytokines. One of the primary compounds involved is vascular endothelial growth factor (VEGF) and platelet-derived growth factor (PDGF), whose intricate regulation involves a plethora of molecular pathways [77]. Hypoxia induced by compounds such as STAT3, Mucin (MUC) 1, NF-κB, and even TAMs with activated CXCL1 and CXCL8 leads to the increased expression of VEGF-A and PDGFB, prompting the formation of tubular endothelial structures that subsequently develop into new blood vessels [137,138,139]. In addition to angiogenesis, the growth of new lymphatic vessels in the TME is also influenced by various factors originating from pancreatic cancer cells and other cells, such as M2-like TAMs [140]. The molecular elements engaged in lymphangiogenesis closely resemble those involved in angiogenesis. Notably, VEGF-C/D emerges as a significant factor in lymphangiogenesis, as its levels rise in individuals with PDAC, which is associated with enhanced invasion of lymphatic vessels, lymph node metastasis, and reduced five-year survival rates [141]. The concentration of CD10+ PSCs, Tregs, and M2-like TAMs is also related to lymphatic metastasis [142,143,144].

## 6. Targeted Precision Therapies

Targeted precision therapies focusing on the TME in pancreatic ductal adenocarcinoma (PDAC) have shown potential, particularly by targeting the stromal components crucial for tumor progression and resistance to treatment. Targeting CAFs can yield both beneficial and detrimental outcomes for patients due to the diverse functions of different CAF subtypes within the TME. One of the initial strategies involved is inhibiting the SHH in CAFs. However, the outcomes were inconclusive; while acute inhibition improved drug penetration into tumors, prolonged inhibition paradoxically promoted tumor progression, highlighting the potential of this approach while leaving the ideal duration of inhibition uncertain [74]. Alternative strategies, such as degrading hyaluronic acid synthesized by CAFs using pegylated recombinant human hyaluronidase 20 (PEGPH20), initially seemed promising for enhancing drug delivery. Nevertheless, clinical trials did not achieve the anticipated outcomes, leading to the discontinuation of further research in this area [82] (Table 2).

### 6.1. Targeting Fibrosis in TME

The utilization of pirfenidone-loaded MMP2-responsive liposomes significantly enhanced gemcitabine penetration to the MMP2-rich pancreatic tumor site in a combinatorial strategy [152]. This approach targeted the fibrotic TME using pirfenidone, an antifibrotic drug that reduces CCL2 and CCL12 production by fibroblasts, which in combination with gemcitabine, reduced tumor volume in mouse models and the levels of tenascin C, fibronectin, and collagen I proteins compared to either gemcitabine or pirfenidone alone [152].

Similar results were observed with losartan, an angiotensin 2 receptor antagonist since it possesses an antifibrotic activity [153]. It has been shown to reduce the activity of TGFβ and stromal collagen, specifically collagen 1, thus increasing drug efficacy and penetration (NCT04106856) (Table 1). Another promising precision target is Focal adhesion kinase (FAK), which is known to promote immunosuppression and fibrosis [154]. It is therefore evident that its inhibition with the use of Defactinib seems like a promising way of reducing desmoplasia, increasing drug delivery and T cell infiltration. This drug is currently being assessed in clinical trials in conjunction with pembrolizumab, a PD-L1 antibody, with promising early results highlighting its ability to overcome radiation resistance on top of its expected effects (NCT03727880 and NCT02546531) (Table 1) [82]. FAP-expressing CAFs produce elevated levels of CXCL12, which hinders the infiltration of T cells into tumors. The removal of these fibroblasts, or the inhibition of CXCL12 and its receptor CXCR4 using inhibitors NOX-A12 and BL-8040, respectively, has demonstrated increased sensitivity of PDAC to immune checkpoint blockade in preclinical studies, thus increasing overall survival (NCT03168139 and NCT02826486) (Table 1). While FAP inhibitors such as UAMC-1110 and talabostat have not shown significant efficacy as standalone treatments, the use of CARs targeting FAP has been effective. This approach hinders the tumor-supporting desmoplastic reaction in animal models [155,156,157]. CAFs also express discoidin domain receptor 1 (DDR1), and its suppression through in murine models significantly reduced the migration of cancer cells, thus increasing overall survival [158]. The expression of the vitamin D receptor (VDR) is elevated while that of lipid storage genes is reduced in CAFs. Treatment with synthetic vitamin D analog, calcipotriol, enhances the expression of lipid storage genes, inhibits EMT and MDSCs and sensitivity to chemotherapy [82]. These promising findings have led to the initiation of clinical trials (NCT02754726) (Table 1). Research has also demonstrated that All Trans Retinoic Acid (ATRA), a compound derived from Vitamin A, hinders β-catenin nuclear localization, and it might also facilitate the degradation of the stroma, potentially improving chemotherapy delivery (NCT03307148) (Table 1) [82]. Lastly, when it comes to drugs targeting CAFs, survival seems to be increasing with the use of Fasudil, which inhibits activated Rho-associated protein kinase (ROCK). ROCK, known for increasing MMP levels and subsequently remodel the ECM to enhance metastasis and reduce tumor permeability to drugs, is a very appealing target since its inhibition is patent-free, making Fasudil a potentially financially accessible drug [82].

### 6.2. Targeting TME Immune Cells

Except for the stromal matrix, immune cell interventions show great promise in precision targeting of the TME. Reducing GM-CSF expression in both mesenchymal stem cell-like cancer-associated fibroblasts and the cancerous epithelial tissue significantly reduced the size of the tumor and eliminated metastases. Clinical trials using cabiralizumab, a monoclonal antibody that blocks the CSF1R receptor, in combination with the inhibitor of PD1 checkpoint nivolumab, have demonstrated effectiveness in treating advanced solid tumors, such as PDAC [82]. Moreover, blocking the body’s own Myc protein induces widespread shrinkage of pancreatic tumors by reducing the penetration of macrophages and neutrophils [159]. A different strategy employs CD40 agonists, which are currently being clinically tested, to transform macrophages into a more immunoenhancing state, thereby promoting CD8 T cell infiltration. The combination of gemcitabine with a CD40 agonist is especially advantageous for patients with a low neutrophil-to-lymphocyte ratio [134,160,161,162]. Another target is galectin-9, whose interaction with dectin-1 on macrophages induces a tumor-promoting M2 phenotype in these cells. Blocking galectin-9 promotes activation of CD4+ and CD8+ T-cells and limits immune suppression and, in turn, cancer progression [163]. Therapeutic approaches with small doses of cyclophosphamide along with GVAX enhance immune responses to PDAC primarily by selectively eliminating Tregs, which are known to suppress immune activity [164]. An alternative method involving immunosuppressive cells within the TME focuses on inhibiting the CSF-1 receptor (CSF-1R), which effectively diminishes TAMs and elevates the transcription of PD-L1 and CTLA-4 genes in animal models, thereby increasing the ratio of CD8+ to CD4+ T cells [165,166]. Regulating chemokines represents an additional therapeutic avenue. A receptor found on myeloid cells, CCR2, binds with CCL2 to attract TAMs. Trials where CCR2 was inhibited with the use of CCX872 and PF-04136309 alongside standard chemotherapy have yielded inconclusive outcomes [74,167]. This is attributed to the compensatory increase in neutrophil recruitment when CCR2 blockade reduces monocyte infiltration into tumors, while inhibition of CXCR2 produces the opposite effect [168]. Consequently, dual inhibition of CCR2 and CXCR2 enhances survival in preclinical models of PDAC [169]. Lastly, another immune-associated obstacle to eliciting an effective adaptive immune response against PDAC is the insufficient presentation of antigens. PDAC tumors contain a relatively low number of dendritic cells (DCs) compared to other cancers, and those that are present are not optimal for antigen presentation [170]. Consequently, boosting both the quantity and functionality of DCs using Fms-like tyrosine kinase 3 ligand could potentially improve the efficacy of immunotherapies including checkpoint inhibitors, especially when paired with a CD40 agonist [170,171,172].

Approaches modulating the tumor metabolism of the TME can serve as an effective therapeutic strategy for PDAC. TAMs have a variety of targets that can be utilized in the context of PDAC. TAMs undermine the efficacy of gemcitabine by releasing deoxycytidine and promote immune suppression by depleting arginine through arginase activity, thereby reducing its availability and impairing the function of cytotoxic T cells [173,174]. They also secrete CCL18, which enhances lactate secretion, facilitating tumor progression and altering the TME, while also driving TAM M2-like polarization of TAMs, thereby suppressing immune responses. Research involving the depletion of TAMs has demonstrated increased sensitivity of PDAC tumors to gemcitabine, decreased immunosuppression, and a reduction in tumor growth rate [14]. Additionally, a method showing clinical promise involves using CD40 antibodies and stimulates TAMs to upregulate MHC II and CD86 expression, initiating an effective anti-tumor immune response and enhancing the responsiveness to chemotherapy [162,175]. Glutamine antagonists together with PD-1 inhibitors also seem to be tumoricidal due to improved nutrient availability and consequent function of CD8+ T cells [176]. Moreover, inhibition of Proline dehydrogenase 1 (PRODH1) using deshydroproline reduced proline utilization by PDAC cells, minimizing tumor size, cell growth, and viability in laboratory experiments [177].

### 6.3. Targeting Epithelial-to-Mesenchymal Transition

Shifting focus to the topic of epithelial-to-mesenchymal transition (EMT), it is important to acknowledge that, although the current data remain inconclusive, emerging evidence indicates a potentially significant link between the TME and EMT in pancreatic cancer. EMT represents a transformation where cancer cells relinquish their intercellular adhesion capabilities, enabling them to breach the basement membrane and initiate metastasis. In mouse models, heightened levels of IL-6/STAT3 and galectin-1 in PSCs are associated with enhanced expression of EMT markers, facilitated through the activation of nuclear factor erythroid 2 (Nrf2) [145,146]. The TME also supports metastasis by enhancing the ability of cancer cells to migrate and invade. After penetrating the tumor capillaries, these cells can travel via portal veins to sites, such as the liver and lungs. This process is facilitated by various compounds. Macrophage inflammatory protein-3α (MIP-3α) produced by TAMs is one of those, since it increases MMP9 expression. This elevation facilitates the invasion of pancreatic cancer cells through collagen IV [147]. Apart from TAMs, CAFs produce abundant palladin, enhancing the modification of the ECM through the control of Cdc42 [148]. The hypoxic environment induced by the TME also significantly contributes to invasion and metastasis by triggering HIF1, which activates genes like *MMP-2*, *NF-κB*, *CX3CR1*, *LOX*, and *QSOX1* [77]. In clinical samples, hypoxia triggers SHH through a mechanism independent of ligands [149]. Apart from the expression of the abovementioned substances, a hypoxic state favors the production of SHH, stimulating PSCs to release elevated amounts of galectin-1, HGF, Periostin, as well as compounds associated with perineural invasion [77]. Finally, the TME plays a crucial role in enhancing the premetastatic niche in PDAC, which is a microenvironment primed by primary tumors to support metastasis formation in distant organs. TAMs release granulin, prompting HSCs to differentiate into myofibroblasts, creating a fibrotic liver environment that fosters metastatic growth [150]. Additionally, MDSCs and neutrophils utilize the CXCR2 pathway to participate in pre-metastatic niche formation, influencing the migration and activation of these immune cells [151]. Ultimately, the TME plays a pivotal role in cancer progression, influencing key aspects and thus highlighting its significance in shaping the course of the disease.

### 6.4. Targeting Exosomes in the TME

Exosomes arise as critical regulators of cancer cells’ pathological features. Exosomes are secreted from cancer cells and contain DNA, RNA, proteins, lipids, as well as other molecules. Through exosomes, these molecules are delivered to targeted cells, among them cells of the TME. Thereby, exosomes are able to regulate communications between T cells, B cells, NK cells, DCs, MDSCs, TAMs, CAFs, endothelial cells, and cancer cells [178]. Since exosomes are directly associated with regulation of the TME immune cells, it is obvious that exosomes can intervene with the tumor’s response to immunotherapy [178]. In vivo experiments depict that PDAC cells establish communication with CAFs and endothelial cells through exosomes. It seems that when exosome secretion is suppressed, it has a repressive impact on angiogenesis, therefore exosomes mediate neovascularization in PDAC. Furthermore, exosomes establish an inter-organ communication with the kidneys, the lung, and the thymus in PDAC [179]. Corroborating findings demonstrate that a subset of miRNAs derived from exosomes secreted by CAFs promote cell growth and migration of the PDAC cells by activating signal transduction pathways which regulate cancer cell proliferation and apoptosis [180]. Association of the exosomes’ cargo with regulation of TME immune cells provides a promising field to enhance efficiency of immunotherapy by inducing the presence of more immune cells within the tumor and reverse immunosuppression. In this context, a bone marrow mesenchymal stem cell exosome-delivery system has been proposed to silence the galectin-9/dectin 1 pathway and also trigger immunogenic cell death [163].

### 6.5. Other Precision TME Targeting Strategies

Further miscellaneous approaches target the TME of PDAC that are broadly applicable and do not selectively impact specific cell types. One of the precision therapies with the most traction involves PD-1/PD-L1 and CTLA-4, which can be inhibited using monoclonal antibodies such as pembrolizumab, nivolumab, and ipilimumab [106,181]. Relying solely on immune checkpoint inhibitors may not effectively treat pancreatic cancer, likely because of the tumor’s low levels of PD-L1 expression, intricate interactions between the tumor and its surrounding stroma, and dense fibrotic environment. Despite its efficacy, combinational therapy should be the choice since standalone immune checkpoint inhibition may prove ineffective, likely due to the tumor’s low PD-L1 expression, intricate tumor–stroma interactions, and desmoplastic nature [77]. Angiogenesis is a hallmark of PDAC that correlates with poor survival, making it a target for therapeutic intervention in PDAC. After multiple failed clinical trials, research shifted from an anti-angiogenic approach to tumor vessel normalization though the blocking of the VEGF–VEGFR2 axis with Semaphorin 3A (SEMA3A) which targets nucleolin [182]. This approach has shown promise in reducing the hypoxic tumor environment, improving drug delivery, and enhancing the infiltration of CD8+ T cells into tumors via ICAM and VCAM and ICAM [183]. While initial results are promising, further research is warranted. The premetastatic niche, which constitutes a supportive microenvironment in metastatic sites, is also an attractive target within the TME. In the case of PDAC, the main metastasis which is found in the liver can be hindered with PDGF, TGFβ1, and FGF2, antibodies or through the diminishing of Ly6G+ or CXCR2 [184]. The inhibition of the latter receptor can also increase the effectiveness of therapy using anti-PD1 [185]. Peripheral nerves, associated with pancreatic tumors, secrete nutrients which enhance tumor growth and can also be targeted in order to decelerate PDAC progression [186,187,188]. Clinical interest was also evident through the inhibition of IL-1R or IRAK4 which minimized the storm’s collagen thus increasing gemcitabine’s efficacy in animal models. IL-1R is under clinical testing to analyze its applicability in pancreatic cancer along with chemotherapeutic agents (NCT02021422). The invasiveness and metastatic behavior of PDAC can also be minimized through the use of TP-0903, which is currently being tested in a clinical environment, or its ability to inhibit AXL (NCT02729298).

Recent studies underline the intrinsic role of the tumor microbiome in pancreatic ductal adenocarcinoma, with a focus on modulating host immune responses in driving patient outcomes. In the study of Riquelme et al., patients with long-term survival had a quantifiably greater diversity of tumor-associated bacteria compared to those with short-term survival. Specific bacteria generally predictive of survival—including *Sachharopolyspora*, *Pseudoxanthomonas*, and *Streptomyces*—have been pinpointed; *Bacillus clausii* further improves this prediction [189]. Specifically, modulation of the immune response by the gut microbiome was evident from improved immune cell recruitment after fecal matter transplantation from long-term survival patients into tumor-bearing mice, which was coupled with reduced tumor growth. This may indicate that by modulating the composition of the PDAC microbiome, one would be able to enhance not only the overall antitumor immune response but also responsiveness to immunotherapies such as anti-CTLA4 and anti-PD-L1, through reducing immunosuppressive populations like Tregs and enhancing immune activation [190,191]. Apart from that, gut bacteria can metabolize enzymes that activate or deactivate chemotherapy drugs, making them more effective but increasing toxicity levels as well. Such bacteria can change the bioavailability of the chemotherapeutic agent through changes in its degree of absorption and distribution, thus enhancing or reducing the effectiveness of the drug [192]. Such promising metabolites produced by gut microbes include trimethylamine N-oxide (TMAO), which has been found to boost anti-tumor immunity against PDAC as well as 3-IAA, which renders PDAC cells more sensitive to chemotherapy [193,194]. Although such research within mouse models has been carried out to investigate how the microbiome acts to influence tumor growth and interact with the immune system, it is very hard to translate these findings into treatment strategies for patients due to large differences in the composition of the microbiome between individuals and across species. This finding suggests that tumor microbiome diversity and specific bacterial signatures could be applied as biomarkers for stratification of patients in clinical trials and, possibly, as therapeutic targets to improve the outcome of PDAC. In that case, future integration of microbiome analysis into clinical practice, like fecal microbiota-based screening for the early detection of PDAC as suggested by Kartal et al., could open new avenues for individualized treatment approaches and new interventions against pancreatic cancer [195].

## 7. Conclusions

In a society where more than 450,000 people die in a year from PDAC, the urgency to develop new technologies that focus on precision targeting is evident. However, this is not an easy task mainly because of the limited accessibility of PDAC tumors due to a dense stroma that surrounds them, acting as a physical barrier and hindering drug delivery. Even if the accessibility was not an issue, the effectiveness of therapeutic drugs would still be limited due to tumor ability to recruit immunosuppressive cells, activate alternate singling pathways, and modify the ECM to adapt and resist the applicable therapeutic approaches. Considering these challenges, a personalized approach on PDAC, where clear stratification criteria based on the specific characteristics of the patients TME are analyzed with the help of advanced imaging techniques, such as multiplex immunohistochemistry, single-cell RNA sequencing, and spatial transcriptomics, seems to be the only viable solution. In this way, health officials can predict a patient’s response to specific therapies and choose the most suitable, minimizing the possibility of unwanted side effects and low survival outcomes. This identification of TME targets is especially important as most patients need combination therapy that affects multiple aspects of the TME simultaneously in order to get treated. Despite all significant strides made in the field of TME targeting in the last years, further improvements are necessary. The focus of research should be the exploration of the molecular and cellular components of the TME which will be useful in identifying both new therapeutic targets as well as more effective drug delivery systems. The TME should be examined not only at the time of the diagnosis of PDAC but throughout their treatment so that biochemical changes happening in response to environmental changes and treatment regimens can be detected. In doing so, healthcare providers will be able to identify exacerbating and alleviating factors, guiding patients to follow a lifestyle and a treatment regimen that increases survival rates. All the above can only be carried out through clinical trials that ensure the patients’ safety. By continuously refining these strategies, we can move closer to achieving effective and personalized therapies that significantly improve the lives of PDAC patients worldwide.

## Figures and Tables

**Table 1:** This table lists selected ongoing clinical trials investigating various therapeutic strategies for pancreatic cancer. The trials explore combinations of immune checkpoint inhibitors, targeted therapies, and chemotherapeutic agents aimed at enhancing treatment efficacy by modulating the tumor microenvironment.

Title of the Trial	Trial Number
Study Assessing Safety and Efficacy of Combination of BL-8040 and Pembrolizumab in Metastatic Pancreatic Cancer Patients (COMBAT/KEYNOTE-202) (COMBAT)	NCT02826486
Olaptesed (NOX-A12) Alone and in Combination with Pembrolizumab in Colorectal and Pancreatic Cancer (Keynote-559)	NCT03168139
Defactinib Combined with Pembrolizumab and Gemcitabine in Patients with Advanced Cancer	NCT02546531
Study of Pembrolizumab with or without Defactinib Following Chemotherapy as a Neoadjuvant and Adjuvant Treatment for Resectable Pancreatic Ductal Adenocarcinoma	NCT03727880
Losartan and Hypofractionated Rx After Chemo for Tx of Borderline Resectable or Locally Advanced Unresectable Pancreatic Cancer (SHAPER)	NCT04106856
A Pilot, Prospective, Non-randomized Evaluation of the Safety of Anakinra Plus Standard Chemotherapy	NCT02021422
First-in-human Study of Oral TP-0903 (a Novel Inhibitor of AXL Kinase) in Patients with Advanced Solid Tumors	NCT02729298
Combination Therapy for Patients with Untreated Metastatic Pancreatic Ductal Adenocarcinoma	NCT02754726
Stromal TARgeting for PAncreatic Cancer (STAR_PAC)	NCT03307148
Danvatirsen and Durvalumab in Treating Patients with Advanced and Refractory Pancreatic, Non-Small Cell Lung Cancer, and Mismatch Repair Deficient Colorectal Cancer	NCT02983578

**Table 2:** This table outlines the key targets and precision strategies explored in the manuscript, focusing on how various components of the TME can be modulated to enhance treatment efficacy and improve patient outcomes in pancreatic cancer. IL-6, interleukin-6; SAT3, signal transducer and activator of transcription 3; Nrf2, nuclear factor erythroid 2-related factor 2; MIP-3α, macrophage inflammatory protein-3α; MMP9, matrix metalloproteinase-9; HIF1, hypoxia-inducible factor 1; SHH, sonic hedgehog; HGF, hepatocyte growth factor; CXCR2/4, C-X-C chemokine receptor type 2/4; FAK, focal adhesion kinase; PD1, programmed cell death protein 1; PD-L1, programmed death-ligand 1; CARs, chimeric antigen receptors; FAP, fibroblast activation protein; DDR1, discoidin domain receptor 1; VDR, vitamin D receptor; CSF1R, colony-stimulating factor 1 receptor; T-reg, regulatory T cells; CCR2, C-C chemokine receptor type 2; CXCR2, C-X-C chemokine receptor type 2; PRODH1, proline dehydrogenase 1; CTLA-4, cytotoxic t-lymphocyte antigen 4; VEGF, vascular endothelial growth factor; VEGFR2, VEGF receptor 2; PDGF, platelet-derived growth factor; TGFβ1, transforming growth factor β1; FGF2, fibroblast growth factor 2; IL-1R, interleukin receptor 1; IRAK4, interleukin-1 receptor-associated kinase 4; TMAO, trimethylamine N-oxide; 3-IAA, 3-indoleacetic acid.

Category	Target Strategy	Mechanism	References
Epithelial-to-Mesenchymal Transition (EMT)	IL-6/STAT3, galectin-1, Nrf2, MIP-3α, MMP9, palladin, HIF1, SHH, HGF, periostin, granulin, CXCR2 pathway	These molecules and pathways are linked to the promotion of EMT, enhancing the invasion and migration abilities of pancreatic cancer cells within the TME	[145,146,147,148,149,150,151]
Targeting fibrosis in TME	Pirfenidone, Losartan, FAK inhibitors (Defactinib), PD-L1 antibodies (Pembrolizumab), CXCR4 inhibitors (NOX-A12 and BL-8040), CARs targeting FAP, DDR1 inhibitors, VDR modulation, ATRA*, Fasudil	Targeting stromal components such as CAFs, fibrosis, and ECM remodeling, using various inhibitors and drugs, to enhance drug delivery, reduce fibrosis, and improve therapeutic outcomes	[82,152,153,154,155,156,157,158]
Targeting TME immune cells	CSF1R inhibitors (Cabiralizumab), PD1 checkpoint inhibitor (nivolumab), Myc blockers CD40 agonists, galectin-9 blockade, T-reg elimination, CCR2 and CXCR2 inhibitors, dendritic cell boosting, glutamine antagonists, PRODH1 inhibition	Strategies focus on modulating immune cells within the TME, enhancing anti-tumor immune responses, and improving therapy sensitivity through various pathways and inhibitors	[14,74,82,134,159,160,161,162,163,164,165,166,167,168,169,170,171,172,173,174,175,176,177]
Targeting exosomes in the TME	Communication via exosomes between PDAC cells and cells of the TME (e.g., disruption of the galectin-9/dectin 1 axis)	Strategies focusing on exploiting the cargo of exosomes against angiogenesis and in favor of tumor efficacy	[163,178,179,180]
Other precision TME targeting strategies	PD-1/PD-L1 and CTLA-4 inhibition (Pembrolizumab, Nivolumab, and Ipilimumab), VEGF/VEGFR2 blockade (SEMA3A), premetastatic niche targeting (PDGF, TGFβ1, and FGF2, antibodies or Ly6G+ or CXCR2 diminishing), IL-1R/IRAK4 inhibition, AXL inhibition (TP-0903), bacterial signatures (TMAO, 3-IAA), targeting microbiota	Broad approaches targeting angiogenesis, immune checkpoint pathways, the premetastatic niche, and other factors within the TME to improve the effectiveness of pancreatic cancer treatments	[77,106,181,182,183,184,185,186,187,188,189,190,191,192,193,194,195]
